# Metropolitan Street Ambulance Association

**Published:** 1906-11-03

**Authors:** 


### METROPOLITAN STREET AMBULANCE ASSOCIATION.

THE Presidents of the Royal Colleges of Physicians and Surgeons respectively have joined the Council of the Metropolitan Street Ambulance Association.

